# Bidirectional Associations Between Highly Processed Food Consumption and Major Depressive Disorder: A Cross-Sectional Study on Adults Residing in Madrid and Barcelona (Edad con Salud)

**DOI:** 10.1192/j.eurpsy.2026.10610

**Published:** 2026-07-31

**Authors:** J. Domènech-Abella, C. Domènech, J. M. Haro, I. Gete-Alejandro, M. Miret, J. L. Ayuso-Mateos, J. Godino-Cruz, E. Lara

**Affiliations:** 1 Fundació de Recerca Sant Joan de Déu, Esplugues de Llobregat; 2 Universidad Autónoma de Madrid; 3 Universidad Complutense de Madrid, Madrid, Spain

## Abstract

**Introduction:**

Highly processed food (HPF) consumption has been associated with increased risk of depression. However, the directionality of this relationship remains unclear. Understanding whether HPF consumption contributes to the development of depression, or if depression leads to greater intake of HPFs, is crucial for designing effective prevention strategies.

**Objectives:**

This study aims to examine the bidirectional association between HPF consumption and MDD in a sample of adults residing in Madrid and Barcelona. Specifically, it explores (1) whether higher levels of HPF intake are associated with greater odds of experiencing MDD, and (2) whether the presence of MDD is associated with higher HPF consumption.

**Methods:**

We analyzed cross-sectional data from 1,378 adults participating in the *Edad con Salud* study, conducted in Madrid and Barcelona in 2023. HPF intake was assessed using the short-form Questionnaire for Highly Processed Food Consumption Frequency (sQ‑HPF) and categorized into tertiles (1 = low, 2 = medium, 3 = high). MDD during the past 12 months was assessed with the Composite International Diagnostic Interview (CIDI). Two weighted regression models were estimated: (1) a logistic regression assessing the association between HPF tertiles and 12-month MDD, and (2) an ordered logistic regression testing whether MDD predicts higher HPF consumption. Both models were adjusted for sex, marital status, age, education, and Functional Comorbidity Index (FCI).

**Results:**

Compared to participants in the lowest HPF consumption tertile, those in the medium tertile showed a non-significant trend toward higher odds of 12-month MDD (OR = 2.76; 95% CI: 0.85–8.95; p = 0.092), while those in the highest tertile had significantly increased odds (OR = 8.74; 95% CI: 3.00–25.53; p < 0.001). In the reverse model, participants with 12-month MDD had 4.39 times greater odds of being in a higher HPF consumption tertile (OR = 4.39; 95% CI: 2.22–8.68; p < 0.001).

**Conclusions:**

Our findings reveal a strong bidirectional association between HPF consumption and MDD. Individuals with higher HPF intake are more likely to experience depression, and those with depression are more likely to report greater HPF consumption. These results highlight the need for integrated prevention strategies that address both dietary habits and mental health.

**Disclosure of Interest:**

None Declared

